# Lightweight and High-Strength Sandwich Panels: Materials, Core Architectures, Manufacturing, Mechanical Performance, Multifunctionality and Future Perspectives

**DOI:** 10.3390/ma19173659

**Published:** 2026-08-28

**Authors:** Yuxin Tang, Yi Song, Qiang Liu, Jianhao Wang, Yifeng Zhong, Xiaopu Chen

**Affiliations:** 1 School of Civil Engineering, Chongqing University, Chongqing 400045, China; 20241601048@stu.cqu.edu.cn (Y.T.); 202516106371@stu.cqu.edu.cn (Y.S.); 202416131358@stu.cqu.edu.cn (Q.L.); 202416021003@stu.cqu.edu.cn (J.W.); 202416021014@stu.cqu.edu.cn (X.C.); 2 Key Laboratory of New Technology for Construction of Cities in Mountain Area, Chongqing University, Chongqing 400045, China

**Keywords:** sandwich panel, lightweight structure, face sheet, face-core interface, honeycomb, lattice, auxetic, TPMS, additive manufacturing, impact, fatigue, multifunctional composite, smart structure

## Abstract

Lightweight and high-strength sandwich panels are used across aerospace, transportation, marine, energy, civil and protective systems because large face separation can provide high flexural rigidity and specific load capacity at low mass. This review treats the panel as a coupled material–structure–interface–process system rather than ranking core topology in isolation. A scoping-review workflow was updated to 24 July 2026 and yielded a curated evidence corpus of 112 total references. The review reports the search and screening logic explicitly, uses descriptive rather than field-wide bibliometric claims, and organizes the synthesis around component roles, governing mechanisms, connection regions, performance requirements and application-driven selection. Conventional foam, honeycomb and corrugated cores are compared with lattice-truss, auxetic, origami/kirigami, hierarchical, bio-inspired, graded and triply periodic minimal-surface (TPMS) architectures. Particular emphasis is placed on face-core and nodal junctions, static and dynamic failure-mode competition, fatigue and environmental degradation, fire/thermal/acoustic functions, additive-manufacturing defects, modelling fidelity, and active/adaptive concepts using shape-memory alloys and piezoelectric actuation. Cross-study numerical envelopes that could not be traced to harmonized test conditions are not used as universal rankings; instead, a mechanism-based screening matrix identifies design strengths, limitations and reporting requirements. The synthesis indicates that engineering deployment depends on preserving intended load paths in the as-manufactured panel, controlling interfaces and defects, validating multi-hazard durability and connections at scale, and integrating multifunctional or adaptive functions without creating new weak links.

## 1. Introduction

The reduction of structural mass is a persistent objective in aerospace, transportation, marine, energy and civil engineering. Lower mass reduces fuel or electricity consumption, extends vehicle range, increases payload, decreases inertial loads and can simplify handling and installation. A successful lightweight solution must nevertheless retain load-carrying capacity, stiffness, stability, durability and safety throughout its service life. Sandwich construction addresses this requirement by placing thin, stiff face sheets away from the neutral plane and separating them with a thicker, low-density core. The faces primarily sustain tensile and compressive stresses generated by bending, whereas the core carries transverse shear, stabilizes the compressed face, transfers local loads and preserves the separation of the skins [1,2,3]. The resulting increase in second moment of area can provide a large gain in flexural rigidity with a comparatively small mass penalty.

The classical concept is simple, but modern sandwich panels are highly heterogeneous structural systems. Face sheets may be metallic, fiber-reinforced polymeric, ceramic, wood-based or hybrid laminates. Cores range from homogeneous foams and expanded honeycombs to folded sheets, lattice trusses, auxetic cells and mathematically generated shell networks. The interface may be an adhesive film, a co-cured resin-rich transition, a welded joint or a continuous additively manufactured connection. Each constituent can become the critical link. A stiff core can shift failure to face wrinkling or debonding; a strong face can increase the demand on the core; a thick adhesive may reduce stress concentration but increase compliance and mass; and an optimized ideal geometry may lose its advantage when manufacturing defects trigger local buckling.

The phrase “lightweight and high-strength” therefore requires a systems interpretation. Absolute strength is not sufficient, because a dense panel may carry a large load while offering poor specific performance. Conversely, minimizing density can create a fragile structure prone to collapse, high defect sensitivity or low residual strength. Appropriate indicators include specific stiffness, specific load capacity, shear rigidity, buckling resistance, indentation resistance, energy absorption per unit mass, crush-force efficiency, residual strength, fatigue life, fire resistance and lifecycle impact [2,4,5]. The most suitable panel is application dependent and is often a Pareto compromise among several competing objectives.

Research has expanded rapidly from conventional foam and honeycomb panels toward architected cellular cores. Pyramidal trusses use axial member forces to obtain high shear efficiency [6]. Foam filling and cellular hybridization delay local buckling and stabilize collapse [7,8,9]. Auxetic cells exploit lateral contraction and rotation mechanisms to redistribute local loads [10,11,12,13,14,15,16,17,18,19,20,21,22]. Origami and kirigami introduce programmable folding paths [23,24,25,26,27,28,29]. TPMS cores provide continuous, smooth surfaces with large internal area and fewer discrete nodes [30,31,32,33,34,35,36]. Additive manufacturing enables these geometries and allows spatial grading, but it also introduces layer anisotropy, porosity, roughness and dimensional deviations [30,31,32,33,34,35,36,37,38,39,40,41,42].

Recent reviews and design studies have addressed energy absorption and protective sandwich structures, including blast-resistant and crashworthy composite sandwich concepts [5,43,44,45], creative core design and materials [46,47], continuous-fiber additive manufacturing [41], additively manufactured cellular structures [42], optimization [48] and foam-core reinforcement [49]. Table 1 makes the distinction explicit. The present review is not intended as an encyclopaedic catalogue. Its specific contribution is an integrated design synthesis that links (i) face sheets, core architecture, interfaces and local junctions; (ii) manufacturing and as-built defects; (iii) governing failure mechanisms under static, dynamic and environmental loading; and (iv) application-level selection, validation and lifecycle constraints. The same framework is then extended from passive load-bearing panels to multifunctional and adaptive sandwich structures.

The distinction is also methodological. Rather than treating topology, material and processing as independent review categories, the present synthesis follows a component-to-performance chain: face sheets and interfaces determine whether a core can mobilize its intended mechanism; manufacturing defects alter the as-built load path; and application constraints determine the relevant normalization and acceptable damage state. This perspective allows mature and emerging concepts to be compared by governing mechanism and system readiness, not by isolated peak properties alone.

**Figure 1:**
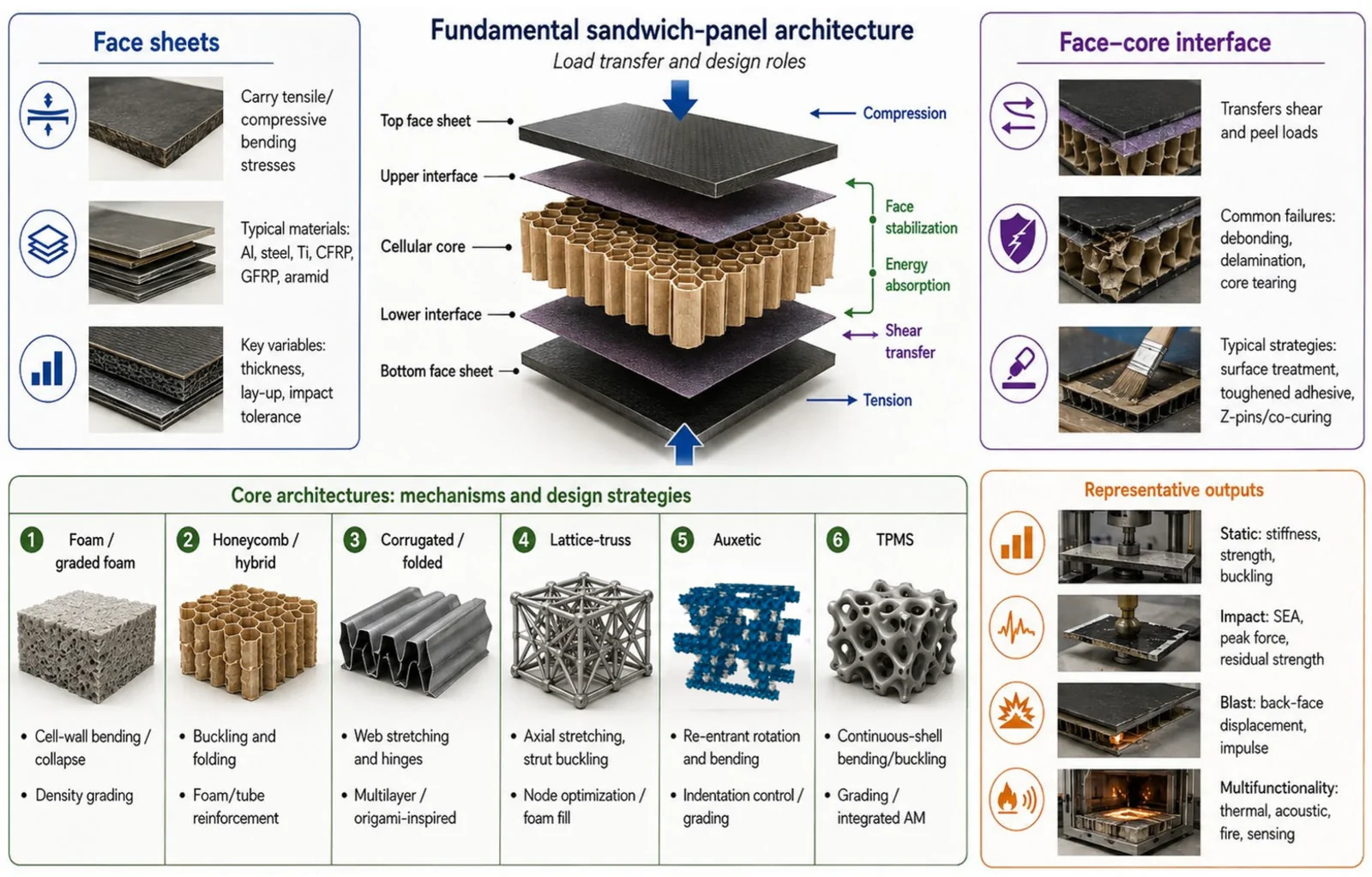
Original schematic of the sandwich-panel system, showing component load paths and representative core families. The figure is intended as the organizing map for Section 4, Section 5, Section 6, Section 7, Section 8, Section 9 and Section 10.

Accordingly, the objectives are to: (1) report a transparent search, screening and evidence-coding strategy; (2) describe the selected literature without inferring database-wide field growth from a purposefully curated corpus; (3) connect face, core, interface and junction choices to governing load paths and failure modes; (4) compare established and architected cores using traceable, mechanism-based criteria rather than heterogeneous numerical rankings; (5) critically compare modelling fidelities and their ability to represent local and progressive damage; and (6) provide an application-driven selection and validation framework that includes multifunctionality, adaptive materials and lifecycle readiness. The component-level architecture and load-transfer logic that organize the remainder of this review are summarized in Figure 1.

The remainder of the review is organized accordingly. Section 4, Section 5 and Section 6 move from fundamental architecture and interfaces to representative core families; Section 7, Section 8, Section 9 and Section 10 examine static, dynamic, durability and multifunctional performance; Section 11, Section 12, Section 13 and Section 14 address modelling, application-driven selection and reporting/validation; and Section 15 and Section 16 delimit the scope and synthesize the principal design implications. This sequence is intended to connect component choice, mechanism, performance and engineering decision rather than simply catalogue publications by topology.

## 2. Review Methodology and Evidence Coding

A structured scoping-review approach was used to cover established sandwich technologies and rapidly developing architected cores. Reporting was revised with reference to PRISMA-ScR principles [50]. The search was updated on 24 July 2026. Sources included Crossref- and OpenAlex-indexed metadata and publisher/indexing searches of ScienceDirect, SpringerLink, Wiley Online Library, Taylor & Francis, Sage, MDPI and PubMed Central. The same conceptual Boolean logic was preserved across sources, with syntax adjusted only for platform-specific field tags and quotation rules.

*Canonical Boolean expression: (“sandwich panel” OR “sandwich structure”) AND (foam OR honeycomb OR corrugated OR folded OR lattice OR truss OR auxetic OR “re-entrant” OR chiral OR origami OR kirigami OR TPMS OR gyroid OR graded OR “bio-inspired” OR “additive manufacturing” OR “continuous fiber” OR interface OR debonding) AND (bending OR compression OR shear OR buckling OR impact OR ballistic OR blast OR fatigue OR vibration OR acoustic OR thermal OR fire OR “electromagnetic shielding” OR “structural health monitoring” OR optimization).*

Eligibility was restricted to peer-reviewed English-language journal articles or authoritative books addressing a structural sandwich configuration and reporting mechanical, manufacturing, durability, functional or optimization evidence. Isolated cellular-material studies were retained only when they were directly used to interpret sandwich-core behavior. Non-structural packaging/cladding reports without mechanical evidence were excluded. Conference papers, theses and preprints were not used as the principal basis for quantitative or mechanistic claims.

**Figure 2:**
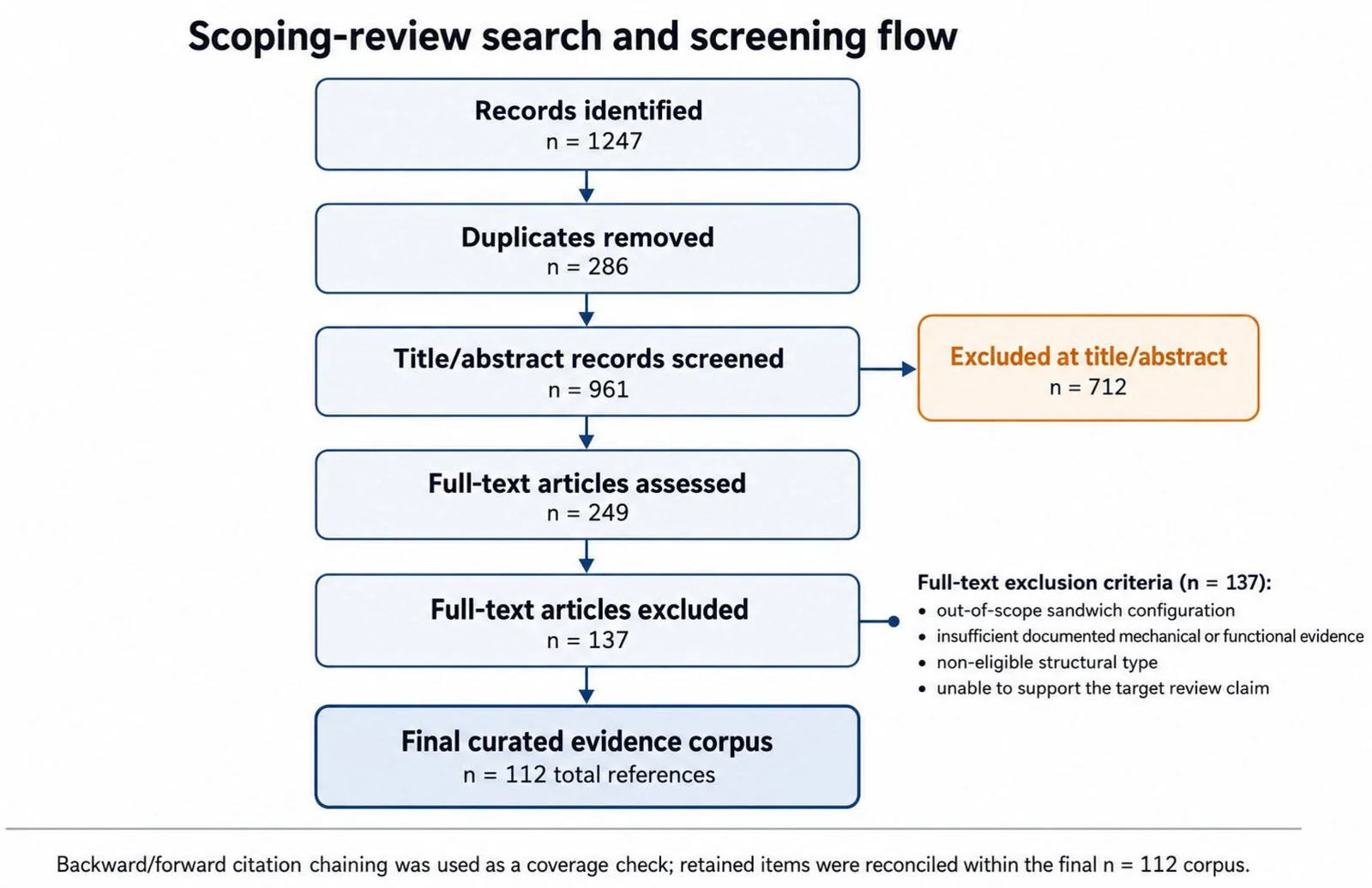
Literature search, screening and evidence-coding workflow. No proprietary database-wide completeness is claimed.

The integrated search identified 1247 records. After removing 286 duplicates, 961 records underwent title/abstract screening; 712 were excluded. Full text was assessed for 249 records, of which 137 were excluded for out-of-scope configurations, insufficient structural/mechanical or functional evidence, non-eligible document type, or inability to support the claim for which the record had been retrieved. The final curated evidence corpus contains 112 references. Backward/forward citation chaining was used as a coverage check and reconciled within the same final corpus. Screening decisions were recorded at title/abstract and full-text stages, and ambiguous cases were resolved by author consensus. The numerical identification, screening and inclusion process is summarized in Figure 2.

No formal study-level risk-of-bias score was applied because the review does not pool effect sizes or estimate a treatment effect. Instead, evidence was coded for core family, face/core material, manufacturing route, loading mode, specimen scale, principal metric, failure mechanism and application; reporting of density/geometry, boundary conditions, replicate/scatter information and as-built defects was also considered when interpreting the strength of a comparison. This choice is a limitation and is explicitly reflected in the avoidance of meta-analytic or universal ranking claims.

For the descriptive title-keyword network, titles of all 112 references were processed using a Python 3.12.4 workflow (pandas/NetworkX/Matplotlib). Full counting was used, the minimum occurrence threshold was two titles, generic stop words were removed, and compound engineering expressions were retained. Predefined synonym merging included “flexural”→“bending”, “3D printed/3D-printed”→“additive manufacturing”, “re-entrant/chiral”→“auxetic”, “truss”→“lattice”, and “gradient”→“graded”. Edge weights are raw within-title co-occurrence counts; no association-strength normalization is used. These plots characterize the selected evidence base only.

## 3. Descriptive Evidence Map of the Selected Corpus

**Figure 3:**
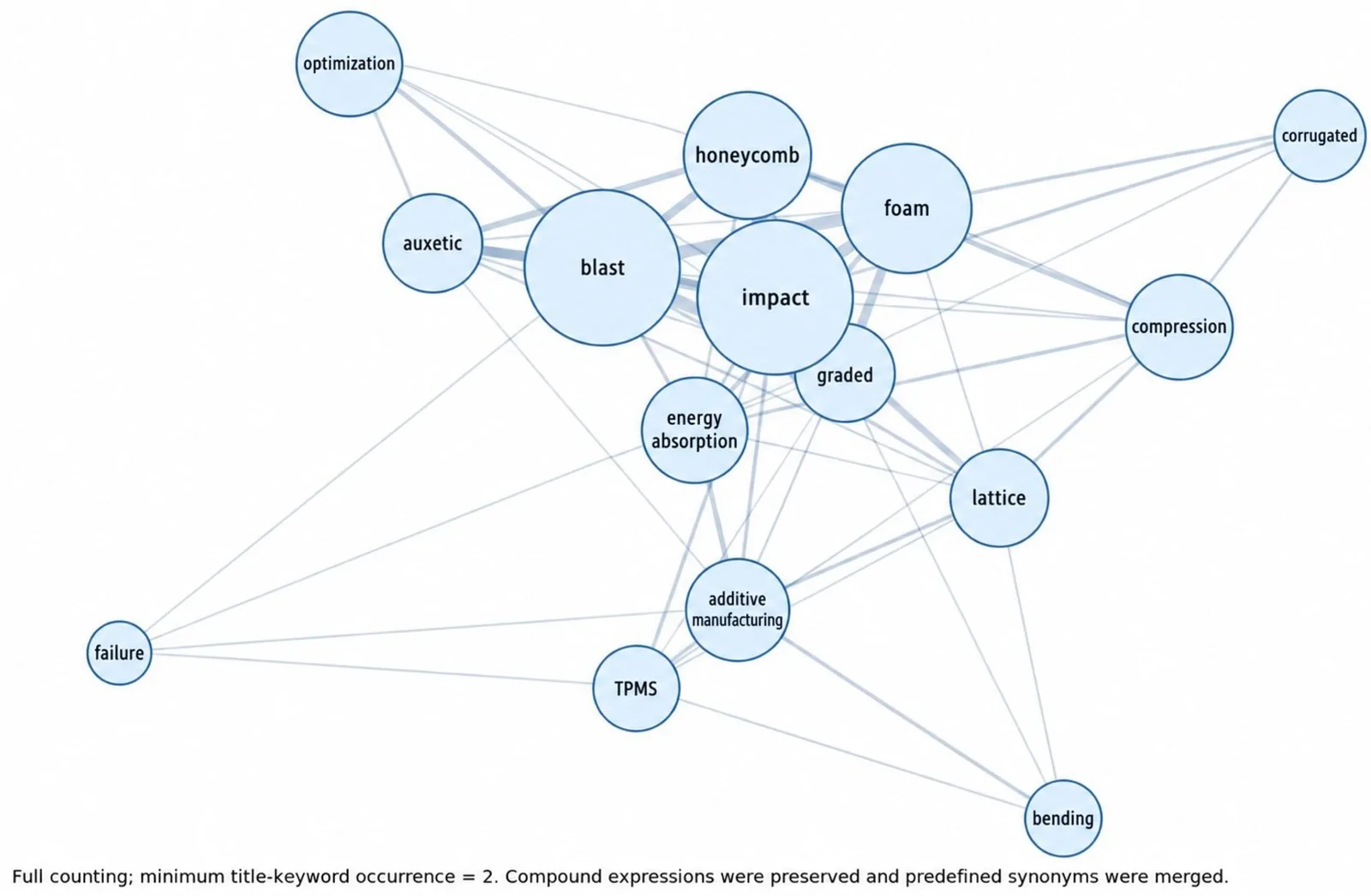
Descriptive title-keyword co-occurrence network for the selected reference corpus (112 total references). Node size represents title occurrence and edge width represents raw within-title co-occurrence.

**Figure 4:**
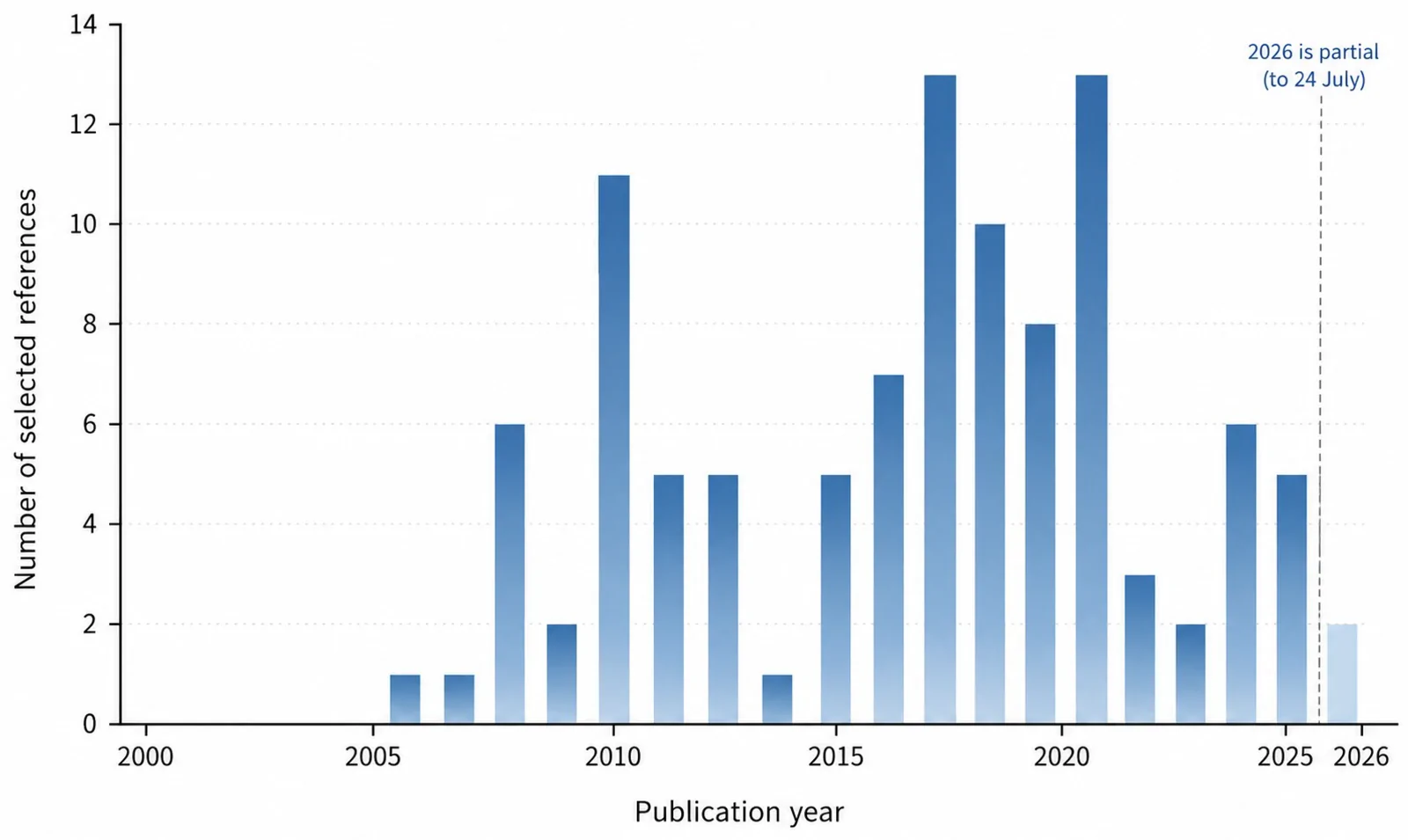
Annual distribution of the selected journal articles. The distribution describes the temporal composition of the curated corpus and should not be interpreted as the evolution of the full field; 2026 is partial through 24 July.

Figure 3 and Figure 4 are descriptive maps of the selected literature rather than bibliometric indicators of global publication growth. The corpus was intentionally assembled to balance established core families, emerging architectures and engineering functions; therefore, annual counts cannot be interpreted as database-wide trends or publication shares. The selected corpus contains 112 references. The three foundational books [1,2,3] are retained for mechanics and terminology but are excluded from the annual journal-publication distribution.

Within this selected evidence base, mature foam/honeycomb studies supply extensive impact, blast, environmental and validated modelling examples, whereas architected-core papers emphasize topology, additive manufacturing and deformation control. Long-term fatigue, creep, moisture, fire-after-impact, full-scale joints and combined mechanical-environmental loading are less consistently represented. This imbalance motivates the later emphasis on junctions, durability, standardized reporting and building-block validation rather than a numerical ranking of publication frequency.

A mechanism-oriented reading of the map suggests three recurring themes: protective loading and energy dissipation; architecture/manufacturing and deformation control; and optimization/multifunctional integration. Their overlap is important because recent architected-core studies increasingly couple geometry with process feasibility and multiple performance objectives. However, this observation describes the composition of the reviewed evidence only and is not used to infer database-wide growth or priority.

## 4. Fundamental Architecture, Mechanics and Performance Metrics

This section summarizes established sandwich and cellular-solid relations for design interpretation; it does not introduce a new constitutive model. The equations are used only to make the assumptions behind the reviewed literature explicit.

For a symmetric sandwich beam of width *b*, face thickness $t_{f}$, core thickness $t_{c}$, and distance *h* between face centroids, an elementary linear-elastic bending rigidity can be written as

(1) $$D=2E_{f}b\left[\frac{t_{f}^{3}}{12}+t_{f}{\left(\frac{h}{2}\right)}^{2}\right]+\frac{E_{c}bt_{c}^{3}}{12},$$

where *D* denotes panel bending rigidity; $E_{f}$ and $E_{c}$ are the Young’s moduli of the face sheets and core, respectively; *b* is panel width; $t_{f}$ and $t_{c}$ are face-sheet and core thicknesses; and *h* is the distance between the face-sheet centroids.

The first bracket contains face self-bending and the much larger parallel-axis term caused by face separation; the last term is core bending. If $t_{f}\ll h$ and $E_{f}\gg E_{c}$, the faces remain bonded without slip, transverse normal stresses are not dominant, and the geometry is symmetric, the separation term dominates and

(2) $$D\approx \frac{E_{f}bt_{f}h^{2}}{2}.$$

The approximation therefore ceases to be reliable when the core is relatively stiff/thick, the bond is compliant or damaged, local indentation is important, or the panel contains strong asymmetry/curvature.

Total transverse deflection contains bending and core-shear components. Slender panels with stiff cores are commonly bending dominated, whereas short spans and compliant cores can be shear dominated. Neglecting shear deformation can therefore overestimate stiffness for low-density foam, honeycomb loaded in a weak direction and thick architected cores. Classical sandwich theory, first-order shear deformation, zig-zag/layerwise approaches and generalized continua trade model simplicity against the ability to resolve through-thickness and local fields.

Material-level indices such as E/ρ and σ/ρ are useful for screening constituent solids, but they should not be conflated with panel-level efficiency. For a complete sandwich panel, more relevant quantities include bending rigidity per areal mass $D/m_{A}$, load capacity per areal mass, absorbed energy per total panel mass, back-face displacement at fixed areal mass/depth, and residual strength after damage. Adhesive, edge closure, inserts, and face mass can dominate thin-core systems even when the core itself has an attractive normalized property. Here, *E* denotes Young’s modulus, σ denotes strength, ρ denotes density (kg·m^−3^), and $m_{A}$ denotes panel areal mass (kg·m^−2^). For energy-absorption studies, EA denotes absorbed energy and specific energy absorption is defined as

$$SEA=\frac{EA}{m},$$

where *m* is the mass of the tested specimen (including face sheets, core and interfaces unless another mass basis is stated explicitly).

Cellular-core scaling is commonly expressed using relative density $\bar{\rho }=\rho_{c}/\rho_{s}$. In ideal open-cell bending-dominated lattices, modulus often scales approximately as

(3) $$\frac{E^{*}}{E_{s}}\propto {\bar{\rho }}^{\;2},$$

and collapse strength as

(4) $$\frac{\sigma^{*}}{\sigma_{s}}\propto {\bar{\rho }}^{\;3/2};$$

in ideal stretch-dominated lattices, stiffness and strength can approach linear scaling with $\bar{\rho }$ [2]. In this notation, $\rho_{c}$ and $\rho_{s}$ are the core and parent-solid densities, $E^{*}$ and $E_{s}$ are the effective core and parent-solid moduli, and $\sigma^{*}$ and $\sigma_{s}$ are the corresponding strengths.

These exponents are idealizations rather than universal laws. Node eccentricity, wall curvature, finite cell count, face constraint, strut waviness, fiber misalignment, roughness, and porosity can introduce bending and change the effective exponent. At low relative density, small thickness errors also produce large percentage changes in load-bearing area.

Failure-mode competition is therefore the practical design framework. Candidate modes include face yielding/tensile rupture, compressive microbuckling and wrinkling, core shear/crushing, web or strut buckling, nodal fracture, indentation, interfacial debonding and global buckling. A balanced design need not activate all modes simultaneously, but it should avoid a premature local weak link that prevents the intended core mechanism from developing [51].

These relations also explain why equal core density does not guarantee equal panel performance. Increasing face thickness can suppress local indentation or wrinkling but may shift failure toward core shear or interface peel; increasing core depth improves bending leverage but can increase shear compliance and the sensitivity of slender webs or struts. The preferred configuration is therefore the one that postpones a premature weak-link failure while satisfying the application-specific constraints on areal mass, depth, stiffness and allowable damage.

## 5. Face Sheets, Interfaces, Connections and Junction Regions

### 5.1. Face-Sheet Materials

Metallic face sheets are attractive where ductility, impact resistance, electrical conductivity, repairability or fire tolerance is important. Aluminum alloys provide a favorable density-strength balance and are compatible with aluminum honeycomb and foam; steel offers high absolute puncture resistance at a mass penalty, and titanium serves demanding thermal/corrosion environments. Under impact, metallic faces dissipate energy through plastic bending, membrane stretching, petalling and tearing, although thin sheets remain susceptible to indentation and wrinkling.

Fiber-reinforced polymer faces provide high specific stiffness and can be tailored by stacking sequence. Carbon/epoxy dominates high-performance applications, glass-fiber composites offer lower cost and impact tolerance, and aramid fibers improve penetration resistance. Compression-side behavior is sensitive to fiber waviness, matrix cracking and microbuckling, while tensile faces can fail by fiber rupture or delamination. Thermoplastic composite faces are increasingly relevant for rapid consolidation, welding and recycling; natural-fiber faces reduce embodied energy but face variability, moisture, flammability and compression limitations [52].

### 5.2. Face-Core Interface

The face-core interface transfers shear and peel. Adhesive joints may fail cohesively within the adhesive, adhesively at an adherend, by tearing a weak core next to the bond line, or by delamination within a composite face. Free edges, inserts, cut-outs, curvature and prior impact damage generate local peel stresses. Film adhesives provide controlled thickness, paste adhesives accommodate gaps, and surface preparation may include abrasion, cleaning, anodizing, chemical/plasma treatment or primers. Excessive cure pressure can crush low-density cores; inadequate pressure produces voids or kissing bonds. Toughened adhesives, graded interlayers, stitching, Z-pins and interlocking features can improve fracture resistance but may introduce new stress concentrations.

Interface demand is strongly affected by stiffness mismatch between the adherends. A very stiff face bonded to a compliant core can create steep shear and peel gradients near edges, inserts and concentrated loads, whereas a thicker or tougher bond line may smooth local stresses at the expense of added compliance and mass. Extreme environments can further modify blast performance by changing polymer stiffness, interface strength and core collapse behaviour. Gupta and Shukla investigated marine foam-core sandwich composites under blast loading at elevated and reduced temperatures, showing that thermal conditions significantly influence energy absorption and failure evolution [53].

### 5.3. Nodes, Transitions, Inserts, Edges and Module Joints

Connection regions should be designed as structural components rather than treated as geometric details. Five recurring classes are: (i) strut-to-strut nodes inside lattice cores; (ii) core-to-face transitions, including adhesive, co-cured, welded and monolithic printed connections; (iii) edge closures; (iv) inserts or potted regions that introduce concentrated loads; and (v) module-to-module joints required for scale-up. These regions experience stress concentration, local bending and eccentric load transfer and can accumulate resin, lack-of-fusion porosity or residual stress. Their governing length scale is often much smaller than the periodic unit cell or global panel.

Nodal geometry is itself a design variable. Iandiorio et al. [54] demonstrated that TPMS-derived smooth fillets at metal cubic-lattice intersections can improve strength-to-weight response by reducing abrupt stress concentration and changing failure localization. The implication for sandwich design is broader than a single lattice topology: fillet radius, curvature continuity, local thickening and graded transition stiffness should be optimized together with cell topology. Similar logic applies to face-core transitions, where a gradual load-transfer region can reduce peel and local bending relative to a sharp stiffness discontinuity.

**Figure 5:**
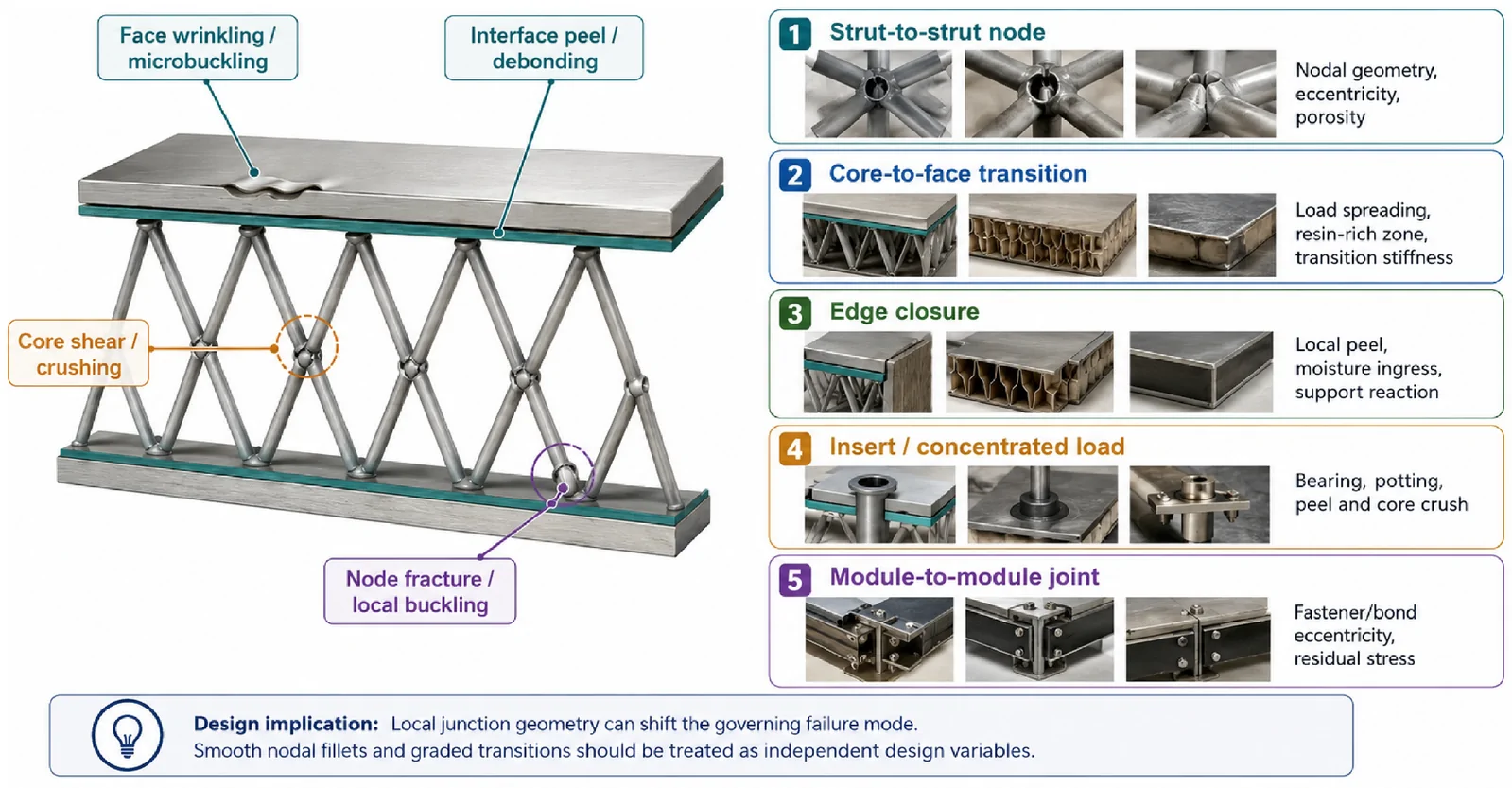
Original mechanics-based map of panel failure modes and connection/junction regions. Local junction geometry, load eccentricity and manufacturing defects can change the predicted failure sequence.

Model fidelity should follow the junction physics. Beam elements can efficiently represent slender strut networks when cross-section deformation and local stress are not governing; shell elements are appropriate for thin-walled cores/faces and many fold or TPMS-sheet models; 3D solids are preferred around thick nodes, inserts, adhesive fillets and contact/bearing regions. Cohesive elements or surface-based traction-separation laws are required when debond propagation is an outcome rather than a prescribed boundary. Local submodels are often the most economical way to resolve a joint while retaining a homogenized global panel. The interacting face, core, node and interface failure modes and representative junction regions are summarized in Figure 5.

## 6. Core Materials and Architectures

Representative internal structures of functionally graded foam, honeycomb/hybrid, corrugated/folded, lattice-truss, auxetic and TPMS cores are illustrated in Figure 1 to support the mechanism-based comparisons in Section 6.1, Section 6.2, Section 6.3 and Section 6.4.

### 6.1. Foam and Honeycomb Cores

Polymer foams provide continuous face support, ease of machining, thermal insulation and compatibility with curved molds. PVC, PET, PMI, polyurethane, polyisocyanurate and SAN foams span wide density and temperature ranges. Core density strongly controls compression plateau stress, shear modulus and energy absorption. Functionally graded foams aim to control collapse sequence: a compliant front layer can reduce the initial impact peak while a denser rear layer supports the back face, although abrupt density interfaces can reflect waves or localize shear [55,56,57,58]. Similar concepts have been extended to metallic honeycomb cores, where layered or graded configurations can tailor crushing progression and improve blast resistance by controlling stress-wave propagation and deformation localization [59]. Metallic foams provide higher temperature resistance and plateau stress but exhibit cell-morphology and bonding variability [60,61,62,63,64,65,66].

Honeycomb remains a benchmark for high out-of-plane efficiency. Aluminum honeycomb combines compression/shear capacity with thermal and fire resistance; aramid-paper honeycomb offers very low mass, corrosion resistance and dielectric behavior; thermoplastic honeycomb supports welding and recycling. Expansion makes honeycomb orthotropic. Under flatwise compression its walls buckle and fold progressively; under shear they distort and may fracture along inclined bands. Local indentation can hide substantial core damage beneath thin faces, and residual strength may be much lower than visible dent depth suggests [67,68,69,70,71,72,73,74]. Hybridization with grids, tubes, foam or corrugation can stabilize cell walls and create multiple load paths [8,75]. Under extreme blast conditions, metallic foam and honeycomb cores exhibit complex interactions between core crushing, face deformation and interface failure. Large inelastic deformation studies have highlighted the importance of core architecture and bonding conditions in determining the final energy absorption response [76].

From a selection perspective, foam and honeycomb represent different support strategies. Foams provide continuous support, damping and easy shaping, whereas honeycombs provide very low density and high directional efficiency. Hybridization can bridge these behaviors, but its benefit should be demonstrated at equal areal mass because reinforcement frequently increases peak load together with density, joining complexity and the number of potential failure interfaces.

### 6.2. Corrugated, Folded and Lattice-Truss Cores

Corrugated cores are produced by folding, stamping, extrusion or composite molding. Triangular, trapezoidal, sinusoidal, X-shaped and bidirectional forms provide directional shear stiffness and open channels for cooling, wiring, drainage or inspection. Web angle, pitch, wall thickness, core height and ridge-to-face connection govern response; typical failures include web buckling, plastic-hinge formation, fracture at folds, ridge debonding and face indentation [7,29,77,78,79,80]. Origami and kirigami use geometric folding rules to program sequential hinges and tunable stiffness [23,24,25,26,27,28,29].

Lattice-truss cores include pyramidal, tetrahedral, octet, Kagome and related networks. Ideal stretch-dominated lattices carry axial strut force, but node eccentricity, face constraint and manufacturing imperfections frequently introduce bending. Composite truss panels can fail by strut buckling/fracture, face crushing or node debonding [6,9,81,82,83,84,85,86,87,88,89]; Metallic lattices dissipate energy through plastic buckling and node rotation. Foam or shear-thickening-fluid filling can support struts against buckling and add rate-dependent resistance [9,89], at the cost of mass, leakage/maintenance and environmental constraints.

For corrugated and lattice systems, the open internal volume is a genuine multifunctional advantage, but it also makes ridge/node quality and face attachment especially influential. A topology classified as stretch dominated at unit-cell level may become bending dominated after face constraint, node eccentricity or manufacturing deviation is introduced. Full-panel validation should therefore confirm that the intended load path survives fabrication and joining rather than relying on the nominal topology classification alone.

### 6.3. Auxetic, Hierarchical, Bio-Inspired and Graded Cores

Auxetic cores exhibit a negative Poisson ratio over part of their deformation range. Re-entrant, chiral/anti-chiral, rotating-unit, double-arrowhead and double-V topologies use wall rotation and bending to contract laterally under compression; under localized loading this can draw material toward the impact zone and increase indentation resistance [10,11,12,13,14,15,16,17,18,19,20,21,22]. The trade-off is that re-entrant walls are often bending dominated, sharp corners concentrate stress, thick faces can suppress in-plane motion, and the auxetic mechanism can disappear after wall contact. Reported advantages should therefore be compared at equal areal mass, comparable faces and consistent boundaries.

Hierarchical cores introduce load-bearing features at multiple scales, for example by replacing a honeycomb wall with a smaller lattice or reinforcing a primary core with grids/tubes [8,68,75]. Hierarchy is useful when the secondary scale delays a governing instability rather than merely adding complexity. Bio-inspired designs abstract mechanisms from bone, bamboo, wood, shells or plant stems; useful transferable principles include anisotropy, gradients, crack deflection and hierarchical interfaces rather than visual imitation. Bio-inspired honeycombs and bamboo cellular panels illustrate stable crushing and low-mass compression [90,91].

Functionally graded cores vary density, wall thickness, cell size, topology or material. Through-thickness grading can tailor impact/blast compaction, whereas in-plane grading can reinforce load-introduction zones, edges and inserts. Continuous grading can reduce stress discontinuities relative to layered designs but increases process-control and characterization demands. The useful design variables include gradient direction and smoothness as well as magnitude.

Grading and hierarchy are most effective when they are aligned with a known stress field or desired collapse progression. A through-thickness gradient that improves a localized impact response can be neutral or detrimental under in-plane shear, reversed loading or a different support condition. For multi-axis service environments, smooth and locally targeted grading is therefore generally more defensible than assuming that a single monotonic density gradient is universally beneficial.

### 6.4. TPMS Cores and Additive Manufacturing

Triply periodic minimal surfaces are mathematically generated periodic surfaces that divide space into interpenetrating domains. Common families include Gyroid, Diamond, Primitive, I-WP and Neovius. Sheet-based TPMS cores use continuous curved walls, avoiding many sharp strut intersections while providing large internal surface area for heat transfer or fluid functions. Their mechanics depend on topology, relative density, sheet thickness, cell size, orientation and face constraint [30,31,32,33,34,35,36]. In sandwich bending, nonuniform shear/compression means that rankings from uniaxial core tests do not necessarily transfer to full beams.

Additive manufacturing further expands the design space of sandwich panels by enabling integrated cellular cores and architected architectures. Three-dimensional printed polymeric sandwich panels have demonstrated promising energy absorption and structural performance, although process-induced defects and anisotropy remain critical challenges [92]. Polymer TPMS can be produced by material extrusion or photopolymerization; metal variants typically use powder-bed fusion. Monolithic or integrated continuous-fiber printing can remove a secondary adhesive step [33], but the face-to-core toolpath transition becomes a new interface containing bead-fusion and consolidation risks. Measured as-built density and geometry should therefore replace nominal CAD values in high-fidelity analysis whenever feasible [35,93]. A mechanism-based comparison of the representative core families discussed above is summarized in Table 2.

Process-aware topology selection is particularly important for TPMS and other additively manufactured cores. Closed or tortuous channels can complicate powder/resin removal and internal inspection, thin walls amplify thickness scatter, and large panels may require modular joining that reintroduces interfaces. Geometric freedom should therefore be evaluated together with minimum feature size, build orientation, post-processing access and the repeatability of the face-core transition.

## 7. Static Mechanical Response and Mechanism-Based Core Screening

Flatwise compression typically exhibits an elastic regime, an instability peak, a plateau and densification. Load-bearing panels may benefit from a high peak/modulus, whereas energy absorbers require a stable plateau and delayed densification. Faces constrain lateral deformation and can increase apparent core strength, so bare-core and complete-panel results are not interchangeable. In-plane compression can trigger global buckling, face microbuckling/wrinkling, core shear or debonding. Three- and four-point bending combine face tension/compression, core shear and local indentation, and changing span-to-depth ratio can deliberately shift the governing mode.

The original cross-study numerical envelope table has been replaced because heterogeneous materials, relative densities, cell dimensions, test directions, terminal strains, strain rates and face constraints make lower/upper bounds appear more comparable than they are. In particular, specific energy absorption from quasi-static compression, drop-weight impact and other terminal strains is not a common metric unless the integration limits and total mass definition are harmonized. Table 3 therefore uses mechanism-based screening criteria and specifies the variables that must be reported before a numerical comparison is defensible.

Mechanism-based screening is useful because it separates intrinsic architectural tendencies from test-specific numerical values. Equal relative density is appropriate for comparing core-level deformation efficiency, equal areal mass and depth are more relevant to panel-level lightweight design, and equal peak force or back-face displacement may be appropriate for protective systems. The normalization condition should therefore be selected before a claim of superiority is made, not after the preferred result is observed.

## 8. Impact, Ballistic and Blast Resistance

Low-velocity impact is a critical threat to composite sandwich panels because a small visible dent can conceal core crushing, delamination and debonding. The front face first bends and contacts the core; local core collapse spreads, followed by matrix cracking/fiber fracture or back-face deformation. Contact force, duration, absorbed energy, dent depth, internal damage and compression-after-impact strength should be interpreted together [67,68,69,70,71,72,73,74]. Face sheets dominate initial contact/penetration resistance, while the core controls support and load spreading. The contribution of face sheets is particularly important because their thickness controls membrane resistance, bending stiffness and load redistribution. Avachat and Zhou demonstrated that facesheet thickness significantly affects the dynamic response of composite sandwich plates subjected to underwater impulsive loading [94]. Smaller honeycomb cells and denser cores can improve indentation support [72,73,74], whereas a compliant core may reduce acceleration at the cost of larger deflection. Graded, auxetic and TPMS cores seek to combine cushioning and back-face support [10,11,12,13,14,15,16,17,18,19,20,21,22,34,55]. Core density remains a key design parameter for impact resistance. Variations in foam density can alter crushing strength, deformation mode and energy absorption capacity under high-velocity impact conditions [95].

Ballistic impact introduces high strain rates, plugging, petalling, fragmentation and possible projectile erosion. Hard metallic/ceramic strike faces spread or erode the projectile while aramid/tough back faces retain fragments; honeycomb/lattice cores maintain separation and absorb energy only if the interface remains engaged. Blast response is governed by pressure history, impulse, stand-off distance and fluid-structure interaction [96]. The front face accelerates, the core compacts and the back face bends/stretches; excessively stiff cores may transmit high impulse even if they resist crushing. Analytical and experimental studies have demonstrated that the blast resistance of sandwich panels is strongly affected by face-sheet stiffness, core compressibility, interface constraint and failure sequence [97,98,99,100,101,102]. Composite sandwich panels with foam cores and stitched or woven composite skins have shown enhanced blast mitigation through combined face deformation and progressive core crushing mechanisms [99,101]. Numerical and theoretical investigations further revealed the importance of fluid–structure interaction and core constitutive behaviour in predicting large deformation under intense blast loading [97,102,103].

Multi-hit and multi-hazard sequences require residual-property assessment. A first impact changes local stiffness and core support; impact-before-blast, blast-followed-by-fire or fatigue-before-impact can therefore shift the later failure sequence [104]. The design objective should specify allowable damage and residual function, not only pristine peak response. Beyond flat panels, curved sandwich structures have attracted attention for aerospace and protective applications. Studies on foam-core curved composite sandwich panels and shallow shells demonstrated that curvature modifies membrane action, load redistribution and blast resistance [105,106].

Loading rate and spatial scale further complicate comparison. Local indentation and drop-weight tests emphasize face-core contact and near-field crushing, whereas distributed blast mobilizes supports, joints, global bending and membrane action. A core optimized for one loading regime may therefore underperform in another. Reporting residual strength and internal damage together with absorbed energy is necessary to distinguish sacrificial energy absorption from damage-tolerant protection. The interaction between blast loading and pre-existing structural states also requires consideration. Wang and Shukla showed that in-plane compressive loading can alter the blast response of sandwich composites by changing deformation compatibility and failure progression [107].

## 9. Fatigue and Environmental Durability

Fatigue can initiate in faces, interfaces, cell walls or nodes. Composite faces accumulate matrix cracking, delamination and fiber damage; metallic cores crack at folds, welds and nodes; bonded interfaces experience mixed-mode cyclic loading even under nominal global bending. Stiffness degradation may be gradual before abrupt final failure. Variable-amplitude spectra, manufacturing defects and moisture should therefore be part of fatigue qualification, especially for wind-energy, marine and transport panels.

**Figure 6:**
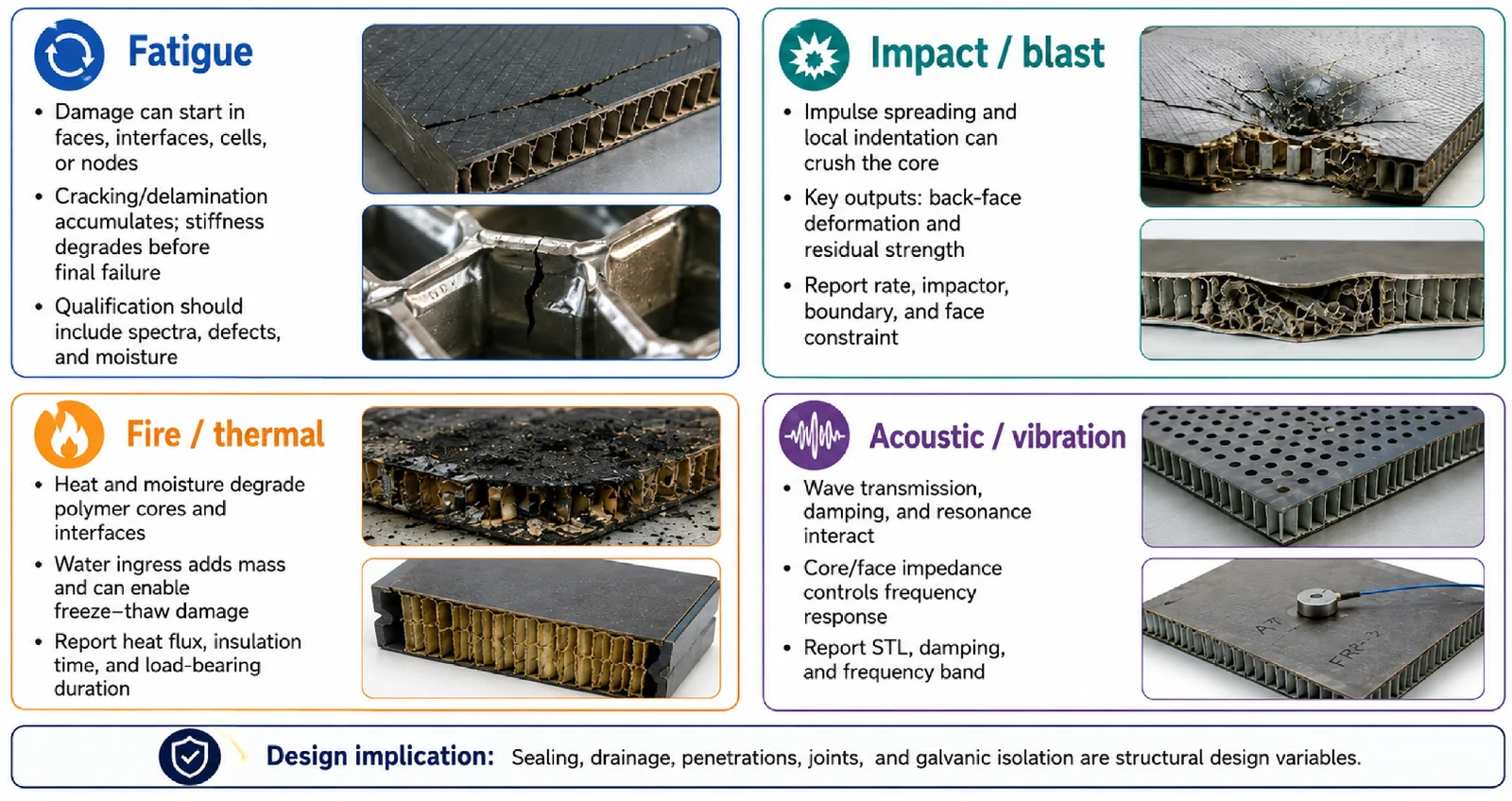
Original schematics linking fatigue, impact/blast, fire/thermal and acoustic/vibration requirements to governing mechanisms and reporting variables.

Polymer cores and adhesives are sensitive to temperature and moisture. Water uptake can plasticize matrices, lower glass-transition temperature and weaken interfaces; damaged honeycombs can retain water through skins/edges, adding mass and enabling freeze–thaw damage. Metallic cores can corrode, and carbon-fiber/aluminum combinations need galvanic isolation. Edge sealing, drainage, penetrations and inspectable joints are structural design variables, not secondary details. The coupled fatigue, impact/blast, fire/thermal and acoustic/vibration mechanisms are summarized schematically in Figure 6.

Durability is often governed by interactions among exposures rather than by a single environment. Moisture can lower adhesive toughness and matrix stiffness and thereby accelerate cyclic crack growth; thermal cycling can amplify mismatch stresses at multimaterial interfaces; and pre-existing impact damage can create fluid-ingress pathways that further weaken the core or bond line. Where service conditions justify it, conditioning should therefore be combined with representative cyclic or residual-strength testing rather than evaluated only as an isolated material exposure.

## 10. Fire, Thermal, Acoustic and Multifunctional/Adaptive Performance

### 10.1. Fire, Thermal, Acoustic and Sensing Functions

Fire is a major limitation of polymeric sandwich systems. Heat softens faces and adhesives, degrades polymeric cores and generates smoke; load-bearing failure can precede loss of insulation. Calcium-silicate/mineral layers, intumescent coatings or hybrid concrete faces can extend resistance. Full-scale GFRP web-core floor tests illustrate the need to assess face rupture, core decomposition, joints and residual stability [108]. Fire-after-impact is especially important because damaged skins and debonds create heat/oxygen pathways.

Acoustic and vibration functions exploit face separation and core volume. Foam adds damping; periodic honeycomb/lattice/TPMS architectures can create resonances or band gaps; local resonators and viscoelastic layers provide frequency-selective attenuation. Foam-filled aramid honeycomb can combine load-bearing support and acoustic insulation [109]. Open lattice/TPMS channels can also function as heat exchangers, but increasing solid fraction often raises both stiffness and conductivity. A design must therefore state whether the intended function is insulation, heat spreading or active convection.

Conductive carbon fibers, metal meshes/coatings and embedded networks can provide electromagnetic shielding and structural health monitoring. Fiber Bragg gratings, piezoelectric transducers and acoustic-emission sensors should be placed to minimize stress concentration and survive core manufacture. Multifunctionality is most efficient when functions share material and geometry rather than being stacked as unrelated layers.

A system-level multifunctional design should quantify both synergy and penalty. Increasing metallic content can improve heat spreading and shielding but add mass and galvanic risk; damping layers can improve acoustic response while changing fire behavior and interface compliance; and embedded sensors may improve observability while creating local stress raisers. The most efficient concepts are therefore those in which a structural feature serves more than one function without introducing a new governing failure mode.

### 10.2. Smart-Material-Enabled and Adaptive Sandwich Structures

Beyond passive multifunctionality, active/adaptive sandwich structures integrate materials that change force, shape or electromechanical state in response to control input. Shape-memory alloys (SMAs) can be embedded in faces, joints or core regions to provide recovery stress, impact-tolerance enhancement or morphing actuation. Li et al. [110] investigated a foam-core sandwich panel with an SMA-hybrid face sheet under low-velocity impact, illustrating how smart reinforcement can be used to modify damage/energy-absorption response rather than acting only as a passive sensor. Testoni et al. [111] demonstrated a modular shape-adaptable sandwich concept with distributed SMA-actuated compliant joints capable of producing in-plane and out-of-plane deformation. These examples show that “smart core” should be defined by a coupled structure-actuator mechanism, not simply by adding a functional material.

**Figure 7:**
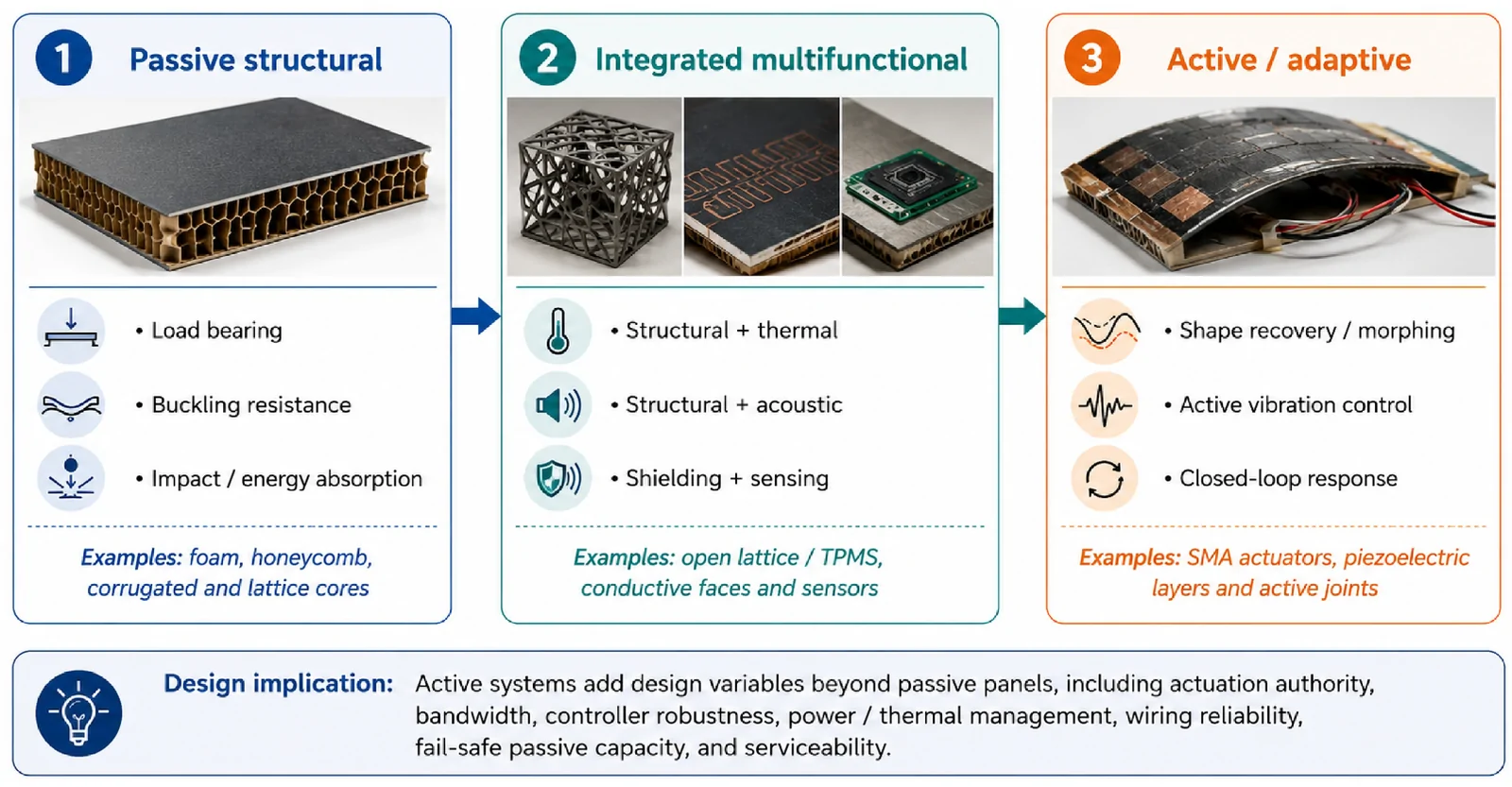
Original classification showing the transition from passive load-bearing sandwich panels to integrated multifunctional systems and active/adaptive smart structures using sensing and actuation.

Piezoelectric layers or patches provide a complementary route for active vibration control because the same electromechanical coupling supports sensing and actuation. Huang et al. [112] investigated active vibration control of piezoelectric sandwich plates, highlighting the additional requirements associated with actuator placement, control law and electrical integration. Active systems therefore add design variables absent from passive panels: actuation authority, bandwidth, power and thermal management, controller robustness, wiring/embedding damage, fail-safe passive capacity and serviceability. The progression from passive load-bearing designs to multifunctional and active/adaptive sandwich systems is summarized in Figure 7.

For active systems, the passive structural baseline remains important. Loss of power, actuator degradation or sensor failure should not remove the minimum load-bearing capacity required for safe operation. Future adaptive-panel studies should therefore report controlled performance together with passive fail-safe response, actuator cycling durability, integration-induced stress concentrations and maintenance/replacement requirements.

## 11. Modelling, Optimization and Data-Driven Design

Analytical models remain valuable for understanding load paths and preliminary sizing. Classical sandwich theory predicts global bending/shear, plastic-hinge and compaction models represent crushing and blast mechanisms, and failure maps compare wrinkling, face failure, core shear and indentation. Their assumptions—homogenized properties, ideal geometry, specified boundary conditions and often perfect bonding—must be stated because they directly determine which failure modes are even possible in the model. A critical comparison of the principal modelling classes and the failure phenomena they can resolve is provided in Table 4.

Boundary conditions and load-introduction regions should be modelled at the fidelity required by the target failure mode. A distributed displacement condition may suppress local bearing or insert failure; perfect clamping can over-stiffen blast coupons; point loads can create artificial singularities. Strain-rate laws are needed when faces, foams, adhesives or lattice collapse are rate sensitive. Geometric imperfections should reflect measured wall/strut deviations or statistically justified fields. Contact definitions control folding, densification and self-contact, while damage regularization or characteristic-length control is required to prevent mesh-dependent energy dissipation in softening models.

As-built tomography, process monitoring and defect descriptors can connect nominal design to reliability analysis. Optimization variables include face thickness/lay-up, core topology and relative density, grading, cell size, adhesive thickness, junction geometry and panel depth. Multiobjective optimization should include manufacturability constraints—minimum wall thickness, overhang, fiber steering, support/powder removal, tool access and inspection—rather than optimizing an ideal CAD geometry. Surrogate and machine-learning models can reduce exploration cost [39], but physics-guided features, uncertainty estimates and explicit extrapolation checks are preferable to unconstrained statistical prediction.

The principal modelling classes are complementary rather than mutually exclusive. A practical workflow can use an equivalent model for global screening, a global–local submodel around an insert, edge or impact zone, and explicit cell/interface models only where local failure governs. Such hierarchical modelling can reduce computational cost while retaining the mechanisms required for validation, provided that constitutive parameters and boundary conditions are transferred consistently between scales.

## 12. Application-Specific Design Selection and Validation

**Figure 8:**
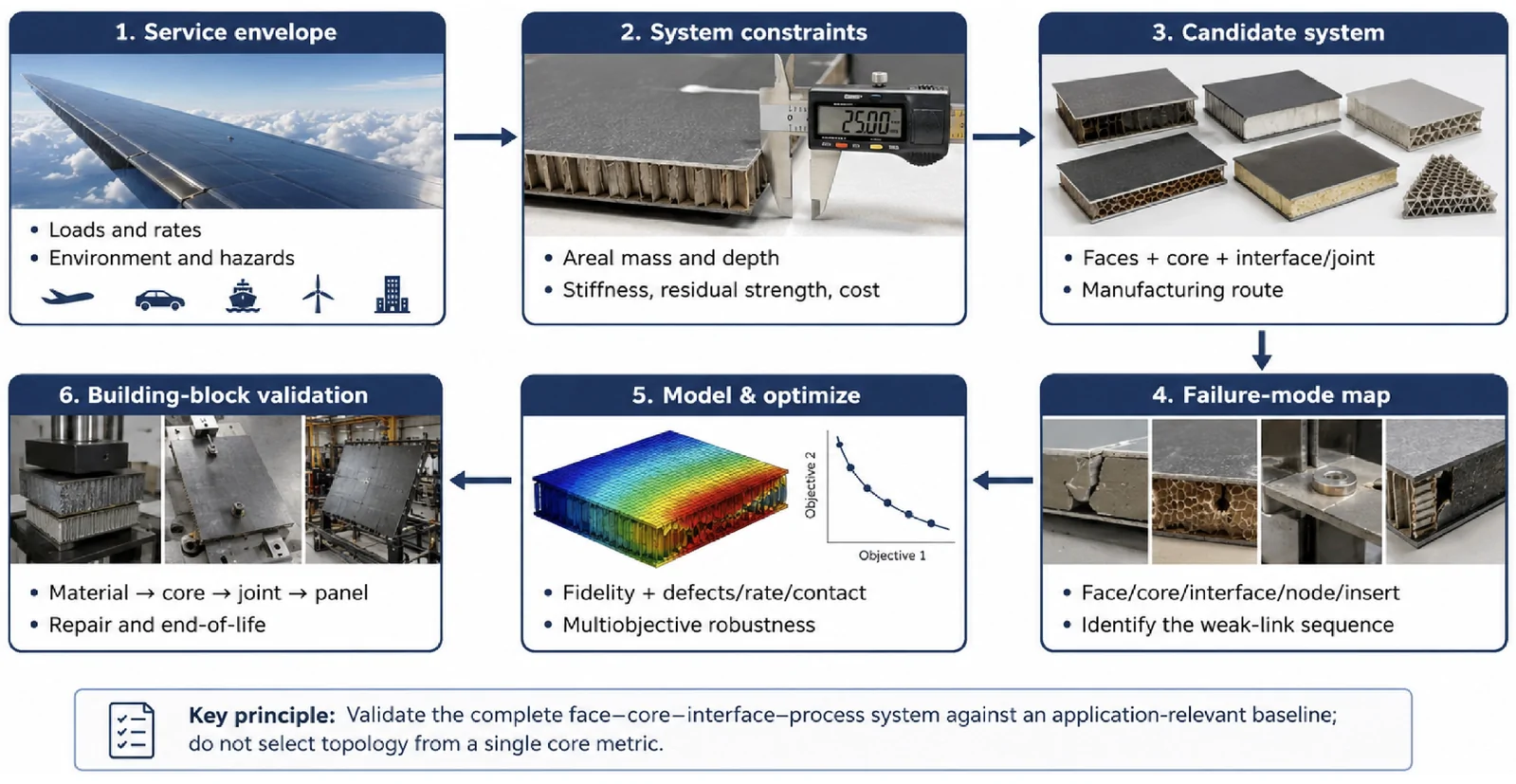
Original application-driven design-selection and building-block validation framework. Candidate topologies are screened only after the service envelope and system-level constraints are defined.

Aerospace panels prioritize specific stiffness, fatigue, dimensional stability, low outgassing, impact tolerance and inspectability. CFRP/Nomex-type honeycomb remains attractive because its behavior and repair routes are established; advanced lattice/TPMS systems must demonstrate defect tolerance and joint/repair methods, not only coupon stiffness. Automotive and rail systems add crashworthiness, cycle time, cost, acoustic comfort and fire safety; battery protection also introduces puncture, thermal-runaway and electrical-isolation constraints. Marine panels require water resistance, fatigue/slamming and fire performance, with edge sealing and penetrations frequently governing service reliability. Civil/building panels must scale economically and satisfy fire/joint requirements [48,90,108]. Wind-turbine sandwich shells/shear webs are dominated by core-shear fatigue, adhesive defects and manufacturing variability. The application-driven screening and building-block validation sequence is summarized in Figure 8.

The practical selection sequence is therefore: define service/load/environmental requirements; choose the governing system constraints (areal mass, depth, stiffness, peak force, residual strength, cost or rate); screen face/core/interface/process combinations by dominant mechanism; construct a failure-mode map including joints and boundaries; select model fidelity and optimize with uncertainty/manufacturability constraints; then validate from constituent and core tests through element/joint, subcomponent and full-scale levels. This prevents selection based solely on an isolated core-compression curve.

Application context also determines which manufacturing disadvantages can be tolerated. Aerospace structures may justify demanding inspection and higher processing cost for small mass savings, whereas rail and building systems may place greater weight on fire performance, repairability and production rate; protective panels may accept sacrificial local damage if residual support and replaceability are maintained. An application-specific decision matrix is therefore more useful than a universal ranking of core topology.

## 13. Research Gaps and Future Perspectives

(1) Interface and junction reliability. Fracture toughness, fatigue and environmental effects must be characterized for new face-core combinations, nodal fillets, graded transitions, inserts and modular joints. The key need is fracture and fatigue evidence under realistic joints, environmental histories and manufacturing tolerances, rather than monotonic coupon strength alone.

(2) As-manufactured defects. Nominal CAD geometry is inadequate for thin-wall AM cores, folded composites and bonded honeycombs; tomography/process monitoring should feed reliability-based models. Defect distributions should be propagated into robustness or reliability analyses so that design decisions reflect manufacturing scatter rather than nominal CAD geometry.

(3) Scale-up and repair. Large panels contain joints, inserts, thickness changes and curved regions. Manufacturing rate, joining, inspection and repair must be reported with mechanical performance. Demonstrators should quantify module-to-module joining, inspection access, replacement strategy and the effect of repaired regions on subsequent load redistribution.

(4) Comparable reporting. Measured density, full geometry, orientation, faces/adhesives, boundaries, replicate scatter, as-built defects and failure images are required for fair baselines. Community-aligned reporting templates and reusable geometry/data files would make cross-study benchmarking substantially more defensible.

(5) Multi-hazard sequences. Impact-fire, blast-fragment, fatigue-impact and moisture-compression combinations should be evaluated using residual strength and retained function. Sequence effects are path dependent, so the same hazards applied in a different order may produce a different damage state and residual capacity.

(6) Hybrid deformation mechanisms. Smooth transitions between stretch-dominated, rotating/auxetic and crushable regions may reconcile service stiffness with extreme-load energy dissipation. The design challenge is to retain service-load stiffness while activating stable rotation, folding or crushing only when the extreme-load threshold is reached.

(7) Physics-guided AI and digital twins. Inverse design should incorporate failure mechanics/process constraints and quantify uncertainty; digital twins should use as-built geometry and in-service sensing rather than nominal CAD alone. Training datasets should include failed or sub-optimal designs and carry explicit uncertainty so that inverse-design recommendations do not simply reproduce publication bias.

(8) Adaptive multifunctionality. SMA/piezoelectric/sensing functions should share structural load paths where possible, while power, control, thermal management and fail-safe passive capacity are treated as system requirements. Benchmarking should compare active performance with the passive baseline and include actuator durability, power demand, control robustness and maintenance burden.

(9) Circularity. Thermoplastic welding, reversible attachment, mono-material architectures, recycled feedstocks and natural-fiber/bamboo systems should be assessed using service life, repair and reuse, not production impact alone. Disassembly, repair and material separation should be considered during architecture and joint design rather than treated only as end-of-life operations.

## 14. Standardized Testing and Reporting Framework

Core-coupon, sandwich-beam and complete-panel tests answer different questions and should not be treated as interchangeable. Core compression identifies through-thickness modulus/peak/plateau/densification; core shear measures load transfer; flatwise tension probes face-core adhesion and near-interface core strength; flexure activates face tension/compression, core shear, indentation and peel; edgewise compression reveals global buckling and wrinkling. Impact, fatigue, fire and environmental tests should be selected from the expected failure map rather than appended as independent demonstrations. Representative sandwich tests and characterization practices are also reported in the experimental literature [30,35,72,74,85].

Representative standardized methods include ASTM C365/C365M-22 [113] for flatwise compression of sandwich cores; ASTM C273/C273M-20 [114] and ASTM C393/C393M-20 [115] for core shear characterization; ASTM C297/C297M-16(2024) [116] for flatwise tensile strength of sandwich constructions; ASTM C364/C364M-16(2024) [117] for edgewise compression; ASTM D7249/D7249M-20 [118] for facesheet properties by long-beam flexure; and ASTM D7766/D7766M-23 [119] for damage-resistance testing of sandwich constructions. These standards provide consistent requirements for specimen configuration, loading, data reduction and reporting, and they should be selected according to the failure mode and design quantity being characterized.

At a minimum, a reconstructable mechanical result should report base-material grade and direction-dependent properties; measured core density and panel areal mass; face thickness/lay-up/fiber volume; cell dimensions and wall/strut orientation; adhesive type, bond-line thickness and cure; manufacturing direction/post-processing; specimen dimensions; loading rate; supports/indenter; conditioning; replicate count/scatter; and the exact definition/integration range of calculated metrics. For AM cores, nominal and as-built wall thickness, porosity, surface condition and minimum feature size should be distinguished. For honeycomb, material type, ribbon direction and cell orientation are essential.

Uncertainty is an engineering result. Mean values without scatter can make variable processes appear competitive with mature systems. Images and internal observations should accompany global curves because similar peak loads can arise from different mechanisms. Numerical models should report mesh sensitivity, constitutive calibration, contact, damage regularization and imperfection definitions, and should be validated against deformation fields, failure sequence and energy partition rather than only a global force-displacement curve.

A building-block validation hierarchy can reduce uncertainty efficiently. Constituent tests calibrate face, core-solid and adhesive properties; core and interface tests identify directional and fracture parameters; element tests reproduce inserts, joints and local load introduction; subcomponents introduce curvature, cut-outs and representative boundaries; and full-scale tests confirm redistribution, progressive damage and inspectability. Calibration and validation should preferably use different datasets so that agreement does not simply reflect parameter fitting to the same experiment.

## 15. Scope and Limitations of the Review

This article is a comprehensive scoping review rather than a database-locked systematic meta-analysis. The search combines broad sandwich-panel terms with targeted architecture, manufacturing, loading and multifunctional terms, and the final evidence corpus was curated to balance foundational mechanics with established and emerging systems. It is not claimed to reproduce every record in Web of Science, Scopus or another proprietary index. Consequently, Figure 3 and Figure 4 describe the selected corpus and do not establish global publication growth, country/institution productivity or citation impact.

The review is restricted principally to the English-language peer-reviewed literature and authoritative books, and publisher/index coverage differs. Title-keyword coding simplifies multidisciplinary articles, and the qualitative screening matrix cannot substitute for a harmonized meta-analysis. The original cross-study numerical ranges were removed because source data do not consistently provide compatible materials, density, geometry, terminal strain, test standard, rate, face constraint and uncertainty. A future quantitative meta-analysis would require datum-level extraction and covariate harmonization.

Publication bias also remains possible: novel high-performing topologies are more likely to be published than failed manufacturing trials or negative comparisons, and small coupons/quasi-static tests are overrepresented relative to full-scale fatigue, fire, repair and lifecycle evidence. The field is developing rapidly, so the stated search date and corpus boundary should be retained when interpreting the descriptive maps.

No formal numerical quality score was assigned to individual studies, so evidence weighting in this review is based on methodological relevance, reporting traceability and consistency with observed mechanisms rather than a single risk-of-bias grade. This is suitable for a heterogeneous engineering scoping review but limits any claim about pooled effect magnitude. The conclusions are therefore conditional and mechanism based, not estimates of a universal performance advantage.

## 16. Conclusions

Lightweight and high-strength sandwich panels achieve structural efficiency by separating stiff faces with a low-density core, but their useful capacity is governed by the coupled material-structure-interface-process system. Conventional foam and honeycomb cores remain competitive because manufacturing, durability and failure behavior are comparatively mature. Corrugated and lattice-truss cores provide directed load paths and open functional volume; auxetic, hierarchical, bio-inspired and graded architectures offer deformation control; and TPMS/additive manufacturing expand geometric freedom and continuous functional integration.

The present synthesis does not support a universal numerical ranking of core families across heterogeneous studies. Meaningful comparison requires application-relevant normalization, consistent boundaries, measured as-built geometry, traceable test conditions, scatter and failure-mode reporting. Local junctions—including nodes, face-core transitions, inserts, edge closures and module joints—can control capacity even when the periodic core itself is strong. Likewise, modelling fidelity must match the target mechanism: global equivalent models are efficient for panel response, whereas progressive crushing, local indentation, junction fracture and debonding require explicit or global–local representations.

Future progress should focus on reliable interfaces/joints, defect-aware design, multi-hazard durability, scalable manufacturing and repair, standardized reporting, physics-guided data-driven optimization and lifecycle circularity. Multifunctional panels should integrate thermal, acoustic, shielding and sensing roles into shared structural geometry, while active/adaptive systems using SMA or piezoelectric actuation should be judged by both structural performance and control/power/fail-safe requirements. The central design question is therefore not which core is “best” in isolation, but which face-core-interface-process architecture preserves the intended load path and required functions throughout manufacture and service.

Overall, the revised review favors a system-readiness perspective: theoretical specific efficiency should be interpreted together with manufacturing yield, joining, inspection, durability, repair and end-of-life options. This provides a common basis for comparing mature foam/honeycomb solutions with architected cores without overstating the significance of isolated coupon metrics.

## Figures and Tables

**Table 1:** Positioning of the present review relative to representative recent review articles.

Review	Year	Materials	Loading	Manufacturing	Interfaces	Quantitative Synthesis	Bibliometric	Main Scope Limitation
Tarlochan [5]	2021	Energy-absorbing sandwich structures; broad core materials	Crash/impact; energy absorption	Limited process discussion	Interface secondary	Yes, performance examples	No formal bibliometric method	Focused on energy absorption rather than integrated system selection
Feng et al. [46]	2020	Foam, honeycomb, corrugated, truss, folded, bio-inspired/hybrid	Mechanical performance; damage	Design/material selection	Discussed at design level	Primarily qualitative	No	Broad creative-design review; limited standardized reporting and life cycle focus
Sahu et al. [47]	2022	Traditional and innovative/smart cores; composite faces	Damage themes	Joining and fabrication emphasized	Joining explicitly discussed	Primarily qualitative	No	Broad advanced-sandwich overview; limited quantitative traceability framework
Cheng et al. [41]	2023	Continuous-fiber 3D-printed lightweight structures	Mechanical properties	Additive manufacturing central	Process transitions relevant	Review synthesis	No	Not specific to full sandwich-system durability and joints
Wu et al. [42]	2023	Additively manufactured materials/structures	Energy absorption/mechanics	AM central	Not sandwich-interface centered	Review synthesis	No	Broader AM scope; limited face-core-system focus
Sada et al. [48]	2025	Composite sandwich structures	Optimization objectives	Manufacturing in optimization	Variable	Optimization-oriented	No	Optimization focus rather than full failure/junction/lifecycle synthesis
Zhang et al. [49]	2026	Foam sandwich core reinforcement	Impact-related reinforcement	Reinforcement routes	Relevant to reinforced core/face transfer	Review synthesis	No	Foam-core reinforcement only
Present review	2026	Conventional + architected cores; metallic/composite	Static, impact/blast, fatigue, fire, thermal, acoustic	Conventional + AM + process qualification	Dedicated interface, node, insert, edge and module-joint treatment	Mechanism-based screening; avoids untraceable pooled ranges	Transparent scoping workflow; descriptive maps only	Integrated material-structure-interface-process-function-lifecycle design framework

**Table 2:** Mechanism-based comparison of representative core families.

Core Family	Dominant Mechanism	Principal Advantage	Principal Limitation	Typical Design Opportunity
Polymer foam	Cell-wall bending/collapse	Continuous face support; insulation; curved manufacture	Creep/moisture; moderate shear; density scatter	Thermal insulation; conventional bonded panels
Metal foam	Plastic cell collapse	Plateau stress; fire/impact tolerance	Irregular bonding surface; mass/scatter	Crash/blast/fire-exposed systems
Honeycomb	Wall buckling/folding; shear distortion	Very low density; mature out-of-plane efficiency	Orthotropy; hidden impact damage; edge sealing	Aerospace/marine/ transport panels
Corrugated/folded	Web stretching/bending; plastic hinges	Directional load paths; open channels	Anisotropy; ridge debonding/fold defects	Transport/protection; functional channels
Lattice-truss	Axial force + strut buckling/node rotation	High specific shear/stiffness; open multifunctional volume	Node defects/joints; inspection complexity	High-performance multifunctional cores
Auxetic	Cell rotation + re-entrant bending	Indentation control; lateral contraction/morphing	Lower initial stiffness in many designs; corner sensitivity	Localized impact/deformation control
TPMS	Continuous shell bending/stretching/buckling	Smooth load transfer; high internal area; grading	AM wall defects; powder/support removal; scale	Thermal/flow integration; AM-enabled grading
Hierarchical/graded/ bio-inspired	Sequential multiscale/spatial collapse	Failure-mode tailoring and redistribution	Process complexity; added defect opportunities	Targeted multi-hazard or local reinforcement

**Table 3:** Mechanism-based screening matrix replacing untraceable cross-study numerical ranges.

Core Class	Intended Mechanism	Common Weak Link	Minimum Information Required for Numerical Comparison
Foam/foam-filled	Continuous support; stable crush	Core shear/crush; face wrinkling; bond failure	Measured density/gradient, cell morphology, temperature/moisture, face/adhesive, terminal strain
Honeycomb/hybrid	High out-of-plane efficiency	Wall folding, shear band, indentation, hidden damage	Material (aluminum vs. aramid-paper), ribbon direction, cell size/wall thickness, panel areal mass, impact geometry
Corrugated/folded	Directional web load paths	Web buckling/hinges; ridge debonding	Web angle/pitch, fold radius, direction, joint detail, span/depth and load introduction
Lattice-truss	Axial load paths with progressive buckling	Node fracture, strut buckling, face-node debonding	As-built strut/node geometry, slenderness, porosity, face constraint, joint manufacturing
Auxetic	Rotational/re-entrant deformation and local load attraction	Hinge/corner fracture; mechanism suppression by faces	Poisson-ratio range, relative density, face constraint, cell count, impact footprint, equal-mass baseline
TPMS	Continuous shell response	Shell buckling/yield; defect-driven localization	Topology definition, measured wall thickness/density, build orientation, roughness/porosity, powder/support removal
Graded/hierarchical	Controlled collapse sequence and local reinforcement	Interface localization or defect accumulation	Gradient law, transition smoothness, local geometry, equal-mass/depth baseline, manufacturing tolerance

**Table 4:** Principal modelling classes for sandwich panels and the failure phenomena they can resolve.

Model Class	Kinematic/Geometric Assumption	Required Inputs	Cost	Typical Scale	Well Suited to Capture	Principal Limitation/Caution
Classical/equivalent single layer	Homogenized face/core; plate/beam kinematics	Orthotropic equivalent stiffness; shear correction; simple failure limits	Low	Panel/global	Global bending, shear, buckling	Poor for local indentation, nodes, debond, as-built defects
Layerwise/zig-zag plate	Piecewise through-thickness kinematic	Layer properties; interfaces; optional cohesive law	Low-medium	Panel/laminate	Interlaminar stress trends; wrinkling with suitable theory	Still homogenizes periodic core unless enriched
Homogenized/micropolar continuum	Periodic unit-cell equivalence; generalized rotations where needed	Effective constitutive tensors from unit cells/tests	Medium	Panel to structural component	Anisotropy, size effects (if generalized)	Local node fracture/crush not directly resolved
Explicit cell-resolved FE	Actual walls/struts/facets; geometric nonlinearity/contact	Solid/shell/beam constitutive and damage rate/contact	High-very high	Coupon/local panel	Indentation, strut/web buckling, progressive crush	Sensitive to mesh, imperfection, contact and damage regularization
Multiscale/global-local submodel	Homogenized global model + local explicit region	Global effective properties + local detailed constitutive data	Medium-high	Panel + critical joint	Efficient joint/insert/node stress and damage	Requires consistent boundary transfer and scale separation
Cohesive-zone interface model	Traction-separation interface with mixed-mode damage	Mode I/II toughness, strengths, mode-mix law	Medium-high	Bond line/transition	Debond initiation/propagation	Parameters need independent calibration; mesh/objective regularization
Surrogate/ML-assisted design	Learned mapping over defined design domain	Curated simulations/experiments + physics-informed features	Low after training; high data generation	Design space	Fast multiobjective screening/inverse design	Extrapolation, uncertainty and causal failure mechanism must be controlled

## Data Availability

No new data were created or analyzed in this study. Data sharing is not applicable to this article.
